# Strong associations of telomere length and mitochondrial copy number with suicidality and abuse history in adolescent depressed individuals

**DOI:** 10.1038/s41380-023-02263-0

**Published:** 2023-09-21

**Authors:** Shinichiro Ochi, Bhaskar Roy, Kevin Prall, Richard C. Shelton, Yogesh Dwivedi

**Affiliations:** 1 Department of Psychiatry and Behavioral Neurobiology, University of Alabama at Birmingham, Birmingham, AL 35294 USA; 2 Department of Neuropsychiatry, Molecules and Function, Ehime University Graduate School of Medicine, Shitsukawa, Toon, Ehime 791-0295 Japan

**Keywords:** Depression, Molecular biology

## Abstract

Major depressive disorder (MDD) is highly prevalent in adolescents and is a major risk factor for suicidality. Recent evidence shows that accelerated cellular senescence/aging is associated with psychiatric illness, including depression, in adults. The present study examined if the relationships of telomere length (TL) and mitochondrial DNA copy number (mtDNAcn), two critical indicators of cellular senescence/aging, are altered in depressed adolescents and whether these alterations are associated with suicidality, early-life adversities, and other co-occuring factors. In genomic DNA isolated from 53 adolescents (ages 16–19, 19 MDD with suicide attempt/suicidal ideation [MDD + SI/SA], 14 MDD without SA/SI [MDD-SI/SA], and 20 healthy controls [HC]), TL and mtDNAcn were measured as the ratio between the number of telomere repeats and that of a single-copy nuclear-hemoglobin [HBG] gene or the amount of mtDNA (NADH dehydrogenase, subunit 1) relative to HBG. Our data show that TL was significantly lower, and mtDNAcn was significantly higher in the total MDD group than HC. TL was significantly lower and mtDNAcn was significantly higher in the MDD + SA/SI group than in the HC, whereas there were no differences in the MDD-SI/SA group. TL was positively correlated with mtDNAcn in both HC and MDD-SA/SI groups; however, TL was negatively correlated with mtDNAcn in MDD + SA/SI. Furthermore, TL was negatively correlated with the severity of both depression and anxiety, while mtDNAcn was positively correlated with the severity of prior emotional abuse. Our study indicates that cellular senescence is more advanced in depressed adolescents with suicidal ideation and that childhood emotional abuse may participate in such a process.

## Introduction

Major depressive disorder (MDD) is the most common psychiatric disorder in adolescents and is significantly associated with increased morbidity [1–3]. In the United States, MDD is also significantly associated with increased mortality [4], and is the most significant biological and psychological risk factor for suicide in adolescents [3, 5–7]. In 2017 the suicide rate in the United States was 11.8 per 100,000 in adolescents and had increased by 57% between 2007 to 2018 [8, 9]. A recent meta-analysis of studies in adolescents showed that the worldwide prevalence of suicide attempts (SA) was 4.6–16.9%, and that of suicidal ideation (SI) was 14.3–22.6% [10]. Given the high prevalence of MDD and suicidality in adolescents, it is not only imperative to elucidate their molecular underpinnings but also to identify the biological markers that are associated with these devastating disorders at early stages and provide strategies for effective interventions.

Accelerated cellular senescence has gained significant attention for its role in stress responsiveness in the last few years [11]. Telomere length (TL) and mitochondrial DNA copy number (mtDNAcn) are the two critical contributors to accelerated cellular senescence [12]. Telomeres are six to eight base pair tandem repeats at the ends of chromosomes that are part of nucleoprotein complexes. They preserve chromosomal stability and integrity by capping the ends of chromosomes, thereby protecting them from degradation or fusion with neighboring chromosomes [13, 14]. Telomeres shorten with each cell division cycle and limit the absolute number of cell divisions. It has been shown that the shortening of TL can cause cellular senescence and DNA damage and is directly associated with age-related diseases, neurodegenerative disorders, and some psychiatric illnesses [13–16]. Interestingly, many [17–23] but not all [24–27] studies suggest a strong association of TL shortening with psychological stress and MDD in adults. Interestingly, higher severity and longer duration of depressive symptoms have been found to be significantly associated with TL shortening [28]. In addition, in a small number of subjects, a few studies showed elevated telomerase activity in MDD patients [29–31], which was significantly correlated with the severity of depression [31]. With insufficient telomerase expression, telomeres shorten gradually with each cell division, which limits the number of times they can divide [32].

MtDNAcn, an indirect measurement of mitochondrial function, is also a marker of cellular senescence and can compensate for mtDNA damage [33]. A few studies have found that people with MDD have more mitochondrial DNA than healthy controls, and there is a relationship between the amount of mtDNA present and previous stress exposure [34]. In a mouse model of depression, it was found that stress significantly increased the amount of mtDNA and decreased telomere length, which was normalized during a four-week recovery period following the stress [35], suggesting that mtDNAcn is regulated via stress. Interestingly, it has been proposed that TL and mtDNAcn can be co-regulated in adult MDD patients via increased hypothalamic–pituitary–adrenal (HPA) axis activity [12, 35]. A recent study also suggests that the two cellular processes are altered in both brain and blood of adults who died by suicide [36].

Reports of TL and mtDNAcn in adolescent MDD patients are very limited. One study found no association between depressive symptoms and markers of cellular senescence; however, depressive symptoms at baseline predicted faster TL shortening and greater increases in mtDNAcn in adolescents who were followed for two years [37]. So far, no study has examined TL and mtDNAcn and their interrelationships with suicidal behavior in adolescent MDD subjects. The present study was undertaken in well-characterized adolescent MDD patients with and without suicidality to examine if: 1) adolescent MDD is associated with aberrations in TL and mtDNAcn; 2) these changes associated with suicidality; 3) the severity of depressive symptoms and/or co-morbid anxiety disorder impact these changes; 4) the changes in TL and mtDNAcn interrelated; and 5) early-life stress (ELS) impact TL and mtDNAcn, and if so, does the type of ELS differentially affect TL and mtDNAcn? We hypothesized that adolescent depressed individuals will show TL shortening and higher mtDNAcn, which will be associated with suicidality. We also hypothesize that early-life stress may mediate these abnormalities.

## Methods

### Participants and clinical assessments

The study was reviewed and approved by the Institutional Review Board of the University of Alabama at Birmingham (UAB). Written informed consent from the parent or guardian and written assent from all participants were obtained before any study procedures. Patients were recruited from the UAB Department of Psychiatry clinics, the UAB Hospital Emergency Department, and the UAB Huntsville Regional Medical Campus. Controls were recruited via advertisement flyers. A total of 53 adolescents (19 MDD with a suicide attempt (SA) or serious suicidal ideation (SI) with intent and plan within two weeks prior to study entry [MDD + SA/SI], 14 MDD without SA/SI (MDD-SA/SI], and 20 healthy non-psychiatric controls [HC]) were included in this study. All participants were evaluated using the Mini International Neuropsychiatric Interview for Children and Adolescents [38]. Depression severity was assessed using the clinician-rated Children’s Depression Rating Scale-Revised (CDRS-R) [39] and the self-rated Beck Depression Inventory (BDI)-II [40, 41]. Anxiety was measured using the Beck Anxiety Inventory (BAI) [42]. History of suicidal ideation, plans, intent, and behaviors was assessed using the Columbia Suicide Severity Rating Scale (C-SSRS) [43]. The history of early life trauma was determined using the Childhood Trauma Questionnaire (CTQ) [44], which yields a total score and subscale score in five areas: emotional (EA), physical (PA), and sexual abuse (SA), emotional neglect (EN), and physical neglect (PN). Longitudinal studies show that PA, SA, and EA are specifically associated with SA risk [45]. A recent meta-analysis also found a markedly elevated risk for SA in people who had experienced PA (OR = 4.11), SA (OR = 3.73), or EA (OR = 3.98) [45]. The patient groups met full DSM-5 diagnostic criteria for MDD [46] and had a CDRS-R score ≥30. Suicidal participants had a C-SSRS score ≥3 rated over the previous two weeks. Controls were free from any lifetime DSM-5 diagnoses. Both males and females were included, and all participants were 21 years or younger. Key exclusion criteria included pregnancy or lactation; being within two months of delivery or miscarriage; any of the following DSM-5 diagnoses or categories: a lifetime history of psychotic disorder; alcohol or drug use disorder (except nicotine/caffeine) within the last month; the use of any hallucinogen (except cannabis), in the last month; bipolar disorder; pervasive developmental disorder; cognitive disorder; DSM-5 paranoid, schizoid, or schizotypal personality disorders (PDs); and anorexia nervosa.

### Collection and processing of blood samples and isolation of genomic DNA (gDNA)

Peripheral blood samples from all participants were obtained and stored at –80 °C before use. gDNA from blood was isolated. Briefly, red blood cell lysis buffer containing 155 mM NH_4_Cl, 10 mM KHCO_3_, and 0.1 mM EDTA was added to the blood and mixed gently. The lysed samples were centrifuged at 6000 rpm x 5 min, and the supernatant was discarded. 500 µl of sodium EDTA was added to the pellet and mixed. The pellet was suspended with 2.5 µl of proteinase-K and 200 µl of SDS and incubated at 55 °C for 2 hours. After incubation, an equal volume of phenol:chloroform:isoamyl alcohol (25:24:1 v/v/v) mixture was added to the samples. After mixing, samples were centrifuged at 13,000 × 20 min at room temperature. The supernatants were transferred to fresh tubes, and 1/10th volume of 3 M sodium acetate, an equal volume of isopropanol, and 1 µl of glycogen were added. After mixing the tube, DNA precipitates were stored at –30 °C overnight. The precipitated DNA samples were centrifuged at 14,000 rpm x 20 minutes at 4 °C. The pellets were washed with 70% ethyl alcohol by centrifuging at 14,000 rpm x 20 minutes at 4 °C. After washing twice, the pellets were dried at room temperature for 10 minutes and were dissolved in Tris-EDTA buffer (pH 8.0). The quality of isolated genomic DNA was initially tested on a Nanodrop to determine the purity of the samples by determining 260/280 and 260/230 ratios for protein and other salt impurities. The DNA samples with values over 1.8 for 260/280 and 2.0 for 260/230 were further processed for qPCR. Later, the samples were also run on a diagnostic gel to identify any RNA contamination besides the presence of high molecular weight genomic DNA.

### Measurements of telomere length and mtDNA copy number

A total of 10 ng of gDNA/sample was used for each measurement. TL was measured using quantitative polymerase chain reaction (qPCR), according to the telomere/single-copy gene ratio method as previously described [36, 47]. Briefly, TL was measured based on the method of determining the ratio between the number of telomere repeats and that of β-globin (HBG), which is a single-copy gene used as a quantitative control, relative to the reference sample. mtDNAcn was determined based on measuring the ratio between the amount of NADH dehydrogenase subunit 1 (ND1) and HBG. All qPCR experiments were performed using Stratagene Mx3005P QPCR System (Agilent Technologies, CA), with EvaGreen® qPCR Master Mix (Low ROX) (Biotium, CA). The primers of telomere were as follows: Forward 5’-GGTTTTT(GAGGGT)^4^GAGGGT-3’ (final concentration 0.27 µM) and Reverse 5’-TCCCGAC(TATCCC)^5^TA-3’ (final concentration 0.9 µM). The thermal cycling conditions for PCR of telomere were: 95 °C for 10 min, 25 cycles of 95 °C for 15 s, and 56 °C for 60 s. The primers for ND1 were: Forward: 5’-AACATACCCATGGCCAACCT-3’ and Reverse: 5’-AGCGAAGGGTTGTAGTAGCCC-3’ (both forward and reverse final concentration 0.8 µM). The thermal cycling conditions of PCR for ND1 were: 95 °C for 10 min, 40 cycles of 95 °C for 15 s, 58 °C for 20 s and 72 °C for 20 s. The primers for HBG were: Forward: 5’-GCTTCTGACACAACTGTGTTCACTAGC-3’ and Reverse: 5’-CACCAACTTCATCCACGTTCACC-3’ ((both forward and reverse final concentration 0.8 µM). The thermal cycling conditions of PCR of HBG were: 95 °C for 10 min, 40 cycles of 95 °C for 15 s, 58 °C for 20 s, and 72 °C for 20 s. The qPCR of all three genes was performed in separate runs using the same samples in the same well positions for each run. The experiments were done in a blinded fashion.

### Statistical analysis

Statistical analyses were performed with SPSS (V.22) software (IBM, Chicago, IL). Shapiro–Wilk test was used for a test of normality. Fisher’s exact test was used for categorical variables. Student’s t-test or ANOVA was used for continuous and ordinal variables, and Bonferroni’s correction was used for *post-hoc* analyses. In addition, to account for the differences in age between groups, ANCOVA was used with age as a covariate. Spearman’s correlation was used for correlation analysis. Multinomial logistic regression was used to test the relationships between categorical and continuous variables In this case, categorical variables were expressed as dummy variables. The significance level was set at *p* ≤ 0.05. The data are expressed as the mean ± standard deviation (SD).

## Results

### Patient characteristics

Detailed demographic and clinical characteristics of controls and MDD subjects are provided in Table 1. There were 40% females and 60% males in the HC group and 79% females and 21% males in the MDD group. The mean age was significantly higher in the MDD + SA/SI group than the MDD + SA/SI (*p* < 0.001) and HC (*p* = 0.007) groups.

**Table 1:** Characteristics of healthy controls and MDD subjects.

	Healthy Controls (HC)	Total MDD	MDD-SA/SI	MDD + SA/SI	*P* value (Comparison between total MDD and HC)	*P* value (Comparison between MDD + SA/SI, MDD-SA/SI, and HC)	*P* value (Comparison between MDD-SA/SI and HC)	*P* value (Comparison between MDD + SA/SI and HC)	*P* value (Comparison between MDD + SA/SI and MDD-SA/SI)
*N*	20	33	14	19					
Female (%)	8 (40.0)	26 (78.8)	11 (78.6)	15 (78.9)	0.0043^**^	0.022^*^	0.12	0.06	1.0
Race (Caucasian:African American:others)	8:11:1	25:5:3	9:3:2	16:2:1	0.007^**^	0.017^*^	0.48	0.022^*^	1.0
Age (years)	16.1 ± 2.7	16.7 ± 2.8	18.8 ± 1.8	15.1 ± 2.5	0.48	0.00026^*^	0.007^*^	0.61	<0.001^***^
BDI-II total	1.7 ± 2.1	28.5 ± 14.0	22.1 ± 13.3	33.3 ± 12.9	<0.001^***^	<0.001^***^	<0.001^***^	<0.001^***^	0.01^*^
CDRS total	2.4 ± 2.7	23.3 ± 12.3	18.2 ± 11.6	26.8 ± 12.0	<0.001^***^	<0.001^***^	<0.001^***^	<0.001^***^	0.04^*^
BAI total	3.0 ± 3.1	24.4 ± 15.1	18.9 ± 14.4	28.4 ± 14.6	<0.001^***^	<0.001^***^	<0.001^***^	<0.001^***^	0.069
C-SSRS (Past week)	NA	2.8 ± 1.5	1.0 ± 0.0	3.3 ± 1.3	NA	NA	NA	NA	<0.001^***^
C-SSRS (Lifetime)	NA	3.4 ± 1.5	2.9 ± 1.8	3.8 ± 1.1	NA	NA	NA	NA	0.13
CTQ total	31.5 ± 10.1	50.2 ± 19.2	45.0 ± 19.6	53.9 ± 18.5	<0.001^***^	<0.001^***^	0.060	<0.001^***^	0.37
CTQ Physical Abuse	7.0 ± 1.8	8.4 ± 4.5	7.8 ± 5.3	8.8 ± 3.9	0.12	0.32	1.0	0.40	1.0
CTQ Physical Neglect	5.7 ± 1.8	7.9 ± 3.3	7.7 ± 4.1	8.0 ± 2.7	0.0034^**^	0.035^*^	0.15	0.04^*^	1.0
CTQ Emotional Abuse	6.2 ± 2.2	13.5 ± 5.6	11.7 ± 6.0	14.8 ± 5.0	<0.001^***^	<0.001^***^	0.0027^**^	<0.001^***^	0.17
CTQ Emotional Neglect	6.7 ± 2.3	12.2 ± 4.9	11.1 ± 5.7	13.0 ± 4.2	<0.001^***^	<0.001^***^	0.0099^**^	<0.001^***^	0.57
CTQ Sexual Abuse	6.0 ± 3.6	8.2 ± 6.6	6.7 ± 5.4	9.4 ± 7.3	0.11	0.16	1.0	0.19	0.56

Values are expressed as mean ± SD except for (%). *P* < 0.05 was considered significant. *p < 0.05, **p < 0.01, ***p < 0.001.

*HC* Healthy Controls, *MDD* Major Depressive Disorder, *SA* Suicide Attempt, SI Suicidal Ideation, *BDI* Beck Depression Inventory, *CDRS* Children’s Depression Rating Scale, *BAI* Beck Anxiety Inventory, *C-SSRS* Columbia-Suicide Severity Rating Scale, *CTQ* Childhood Trauma Questionnaire, *NA* Not Applicable.

The CDRS and BDI-II mean scores in the total MDD group was significantly higher than the HC group (both *p* > 0.001). Similarly, the total CTQ score in the MDD group was significantly higher than the control group (*p* < 0.001). When CTQ subscale scores in five areas of early life stress were considered, physical neglect (*p* = 0.0034), emotional abuse (*p* < 0.001), and emotional neglect (*p* < 0.001) but not physical or sexual abuse were significantly higher in the MDD group than in the HC group.

The BDI-II and the CDRS total scores were significantly higher in the MDD + SA/SI and MDD-SA/SI groups than in HC (*p* < 0.001). MDD + SA/SI group showed significantly higher CDRS total scores than the MDD-SA/SI group (*p* = 0.04). C-SSRS (past week) was also significantly higher in the MDD + SA/SI group compared to MDD-SA/SI group (*p* < 0.001).

The CTQ total score was significantly lower in the HC than the MDD + SA/SI group (*p* < 0.001) but not in the MDD-SA/SI group, although it barely missed significance (*p* = 0.06*)*. When individually measured, CTQ for physical neglect was significantly higher in MDD + SA/SI group than in the HC group (*p* = 0.04), whereas it was not significantly different between MDD-SA/SI and HC groups. On the other hand, both MDD + SA/SI (*p* < 0.001) and MDD-SA/SI (*p* = 0.0027) groups showed significantly higher CTQ for emotional abuse than the HC group. As with physical abuse, CTQ scores for emotional neglect were higher in both MDD + SI/SA (*p* < 0.001) and MDD-SI/SA (*p* = 0.009) groups than the HC group; however, it was significantly lower in MDD-SA/SI group than the MDD + SA/SI group. CTQ score for sexual abuse was not significantly different between MDD + SA/SI, MDD-SA/SI, and HC groups.

### Telomere length and mtDNA copy number between healthy control and MDD groups

**Fig. 1:**
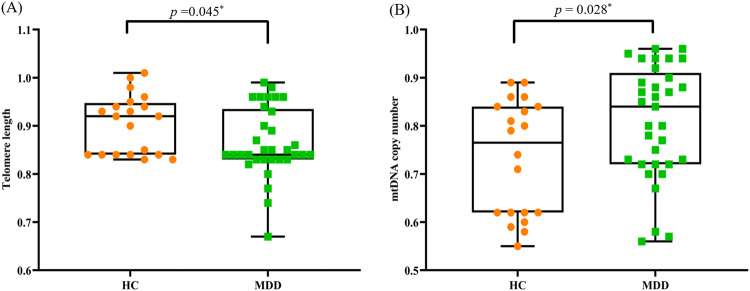
Telomere length and mtDNA copy number between healthy controls and MDD. **A** TL in the total MDD (n = 33) was significantly lower than in the HC (n = 20). **B** mtDNAcn in the total MDD (n = 33) was significantly higher than HC (n = 20). Error bars show the mean ± SD. Student’s *t* test was used. *P < 0.05 was defined as significance. MDD Major Depressive Disorder, HC Healthy Controls, mtDNAcn mtDNA copy number.

Comparisons of TL and mtDNAcn between MDD and HC subjects are shown in Fig. 1. TL in the total MDD group was significantly lower than the HC group (*p* = 0.045) (Fig. 1A). In contrast, mtDNAcn was significantly higher in the total MDD group when compared with the HC group (*p* = 0.028) (Fig. 1B).

To test the possibility of an interaction of TL and mtDNAcn, TL was contrasted between the total MDD and HC groups using ANCOVA with mtDNAcn as a covariate. The TL comparison remained significant (F = 7.31, *p* = 0.009). The opposite interaction was also tested. mtDNAcn was compared between groups by ANCOVA with TL as a covariate and remained significant (F = 8.22, *p* = 0.006). The correlation of TL and mtDNAcn was also non-significant for the total sample (MDD and HC) (r = 0.17, *p* = 0.17) and within the MDD group (r = 0.001, *p* = 0.995). However, TL and mtDNAcn exhibited a strong correlation within the HC (r = 0.84, *p* < 0.001) and MDD-SA/SI (r = 0.54, *p* = 0.045) groups, but not the MDD + SA/SI group (r = –0.35, *p* = 0.14). This indicates that depressed patients show the typical positive relationship between TL and mtDNA found in the controls. However, this expected relationship is lost in the suicidal depressed participants.

### Telomere length and mtDNA copy number between healthy controls and MDD subjects with or without suicidal ideation or behavior

**Fig. 2:**
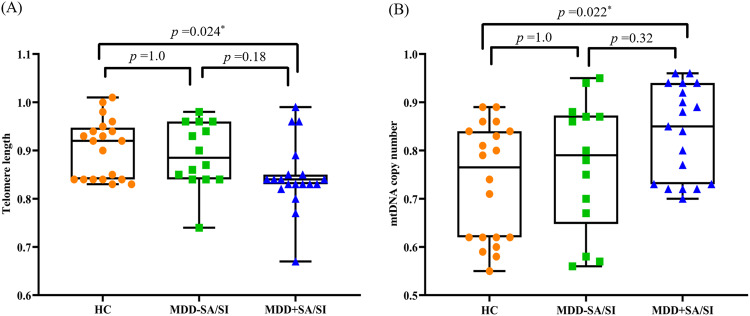
Telomere length and mtDNA copy number between healthy controls, MDD-SA/SI, and MDD + SA/SI. **A** TL in the MDD + SA/SI group (n = 19) was significantly lower than the HC group (n = 20). There was no significant difference between the MDD + SA/SI group (n = 19) and MDD-SA/SI (n = 14) groups and between the MDD-SA/SI group and HC group. **B** mtDNAcn in the MDD + SA/SI group was significantly lower compared to the HC group. There was no significant difference between MDD + SA/SI group and MDD-SA/SI group and between the MDD- SA/SI group and the HC group. Error bars show the mean ± SD. ANOVA adjusted by Bonferroni correction was used. *P < 0.05 was defined as significance. MDD Major Depressive Disorder, SA suicide attempt, SI suicidal ideation, HC Healthy Controls, mtDNAcn mtDNA copy number.

Figure 2 shows the comparisons of TL and mtDNAcn between MDD + SA/SI, MDD-SA/SI, and HC groups. ANOVA showed that both TL (*F*(2, 50) = 4.1; *p* = 0.023) and mtDNAcn (*F*(2, 50) = 4.0; *p* = 0.025) were significantly different between groups. Since age significantly differed between MDD + SA/SI, MDD-SA/SI, and HC groups, we also compared the group differences for TL and mtDNAcn using ANCOVA using age as a covariate. The overall ANCOVA showed that both TL (*F*(2, 49) = 2.9; *p* = 0.042) and mtDNAcn (*F*(2, 49) = 3.3; *p* = 0.026) were significantly different between the three groups. Post-hoc analysis showed that TL in the MDD + SA/SI group was significantly lower than the HC group (*p* = 0.024), whereas MDD-SA/SI was not significantly different from the HC (Fig. 2A). Similarly, mtDNAcn in the MDD + SA/SI group was significantly higher than the HC group (*p* = 0.022) and the MDD-SA/SI was not different from HC (Fig. 2B).

### Correlation between telomere length and mtDNA copy number in each individual group

**Fig. 3:**
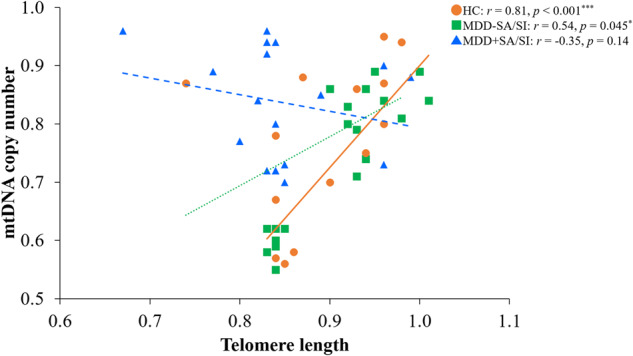
Correlation of Telomere length and mtDNA copy number between healthy controls, MDD-SA/SI, and MDD + SA/SI. TL was significantly positively correlated with mtDNAcn in the HC group (n = 20) and the MDD-SA/SI group (n = 14). Telomere length was negatively correlated with mtDNAcn in MDD + SA/SI group (n = 19), although the data did not reach the significance level. Spearman’s correlation was used. *P < 0.05 was defined as significance. *p < 0.05, **p < 0.01, ***p < 0.001. MDD Major Depressive Disorder, SA suicide attempt, SI suicidal ideation, mtDNAcn mtDNA copy number.

As shown in Fig. 3, TL was significantly positively correlated with mtDNAcn in HC (*r* = 0.81; *p* < 0.001) and MDD-SA/SI (*r* = 0.54; *p* = 0.045) groups. Conversely, TL was negatively correlated with mtDNAcn in MDD + SA/SI group, although it was not statistically significant (*r* = −0.35; *p* = 0.14) (Fig. 3).

### Effect of demographic and clinical variables on telomere length and mtDNA copy number

**Fig. 4:**
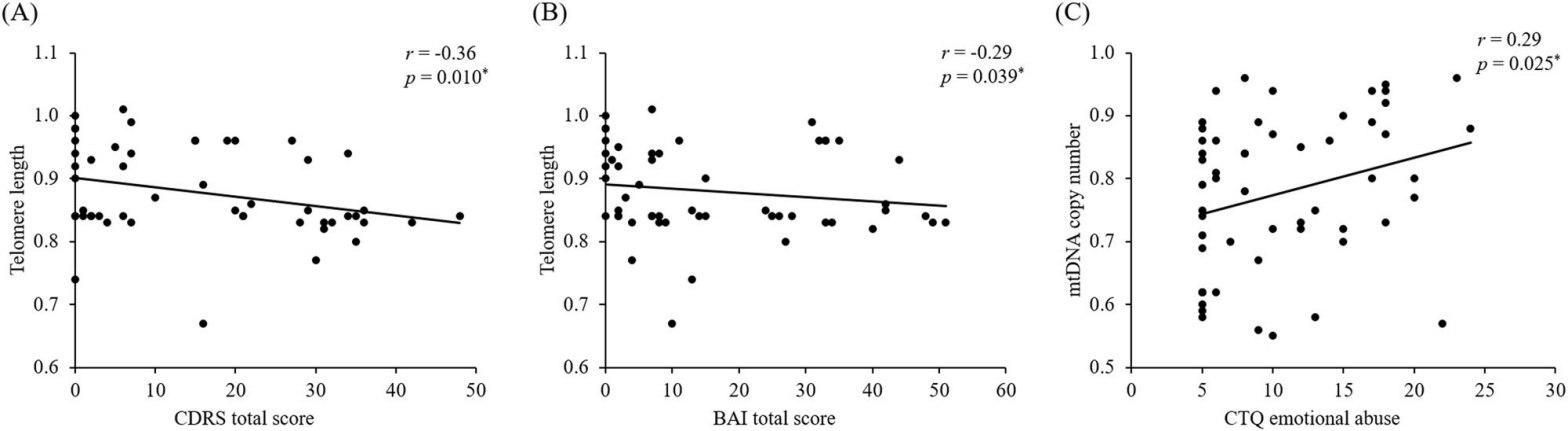
Telomere length and mtDNA copy number between healthy controls, MDD-SA/SI, and MDD + SA/SI. **A** TL was significantly correlated with CDRS total score. **B** TL was significantly correlated with BAI total score. **C** mtDNAcn was significantly correlated with CTQ emotional abuse (EA). *P < 0.05 was defined as significance. *p < 0.05, **p < 0.01. mtDNAcn mtDNA copy number; CDRS Children’s Depression Rating Scale, BAI Beck Anxiety Inventory, CTQ Childhood Trauma Questionnaire.

Correlation of TL or mtDNAcn with clinical and demographic characteristics, including age, suicide severity, BDI-II, CDRS, BAI, C-SSRS (past week), C-SSRS (lifetime), total CTQ in each individual category (CTQ PA, CTQ PN, CTQ EA, CTQ EN, and CTQ SA) were determined. TL, but not mtDNAcn, was significantly negatively correlated with CDRS total score (*r* = −0.36; *p* = 0.010), and BAI total score (*r* = −0.29; *p* = 0.039) (Fig. 4A, B). Multinomial logistic regression indicated that mtDNAcn (Likelihood ratio = 157.2, df=60, *p* = 0.02) but not TL (Likelihood ratio = 107.1, df=60, *p* = –0.20) were associated. However, when age was entered as a covariate, the finding with mtDNAcn became non-significant (Likelihood ratio = 186.2, df=60, *p* = 0.28).

mtDNAcn was significantly correlated with CTQ EA score (*r* = 0.29; *p* = 0.025) (Fig. 4C) but not with other CTQ scores. No significant correlations were observed between other variables and TL and mtDNAcn (Supplementary Table 1, Supplementary Fig. 1, and Supplementary Fig. 2). All the correlations related to TL or mtDNAcn are shown in Supplementary Table 1.

The significant association between EA and mtDNAcn raises the possibility that the differences in the overall depression sample and the MDD + SA/SI subgroup from HC in this variable could be driven primarily by the history of abuse. To test this, the analyses for the MDD versus HC and the subgroups versus HC was repeated using ANCOVA with the CTQ EA score added as a covariate. In fact, the differences for both the overall depression group and the MDD + SA/SI subgroup became non-significant (F = 0.269, p=NS, F = 0.563, p=NS, respectively).

## Discussion

The main findings of this study were that TL was significantly reduced and mtDNAcn was significantly increased in depressed adolescents. The results are consistent with findings in most studies of adults with MDD for both TL [17–23] and mtDNAcn [34]. One prior study in adolescents no association between depressive symptoms and markers of cellular senescence and baseline but did find faster TL shortening and greater increases in mtDNAcn over two years [37]. These results coupled with ours indicate that MDD patients may be experiencing accelerated cellular senescence across the lifespan. Accelerated senescence is associated with a wide variety of stresses, including endogenous characteristics, such as oxidative stress and inflammatory markers such as cytokines and chemokines [48], but also exogenous factors like psychological stress [49]. Accelerated cell aging is associated with increased risks for certain cancers, cardiovascular disease, diabetes and metabolic syndrome, and other conditions [49]. Shortened TL can be transmitted intergenerationally by direct germ line transmission of shortened telomeres and by enhanced cellular stress in utero [49]. The association of depression with accelerated cell aging could explain the increased susceptibility of depressed people to a range of conditions, including metabolic disease, type II diabetes, and cardiovascular disease [50].

An interesting observation in the study was that both the HC and the MDD-SA/SI groups showed positive correlations between TL and mtDNAcn (r = 0.81 and 0.54 respectively), indicating that greater TL was associated with higher mtDNAcn. This relationship was completely lost in the MDD + SA/SI group (r = −0.35). These results suggest that, while both TL and mtDNAcn are significantly different in the MDD versus HC comparison, the mechanisms accounting for each are likely to be different. It also indicates that there may be unique biology in the MDD + SA/SI group that accounts for the lack of relationships between the variables.

Unique to this study was the observation that the differences between MDD patients and HC of both TL and mtDNAcn was predominantly found in MDD patients with acute serious suicidal ideation or behavior **(**Fig. 2**)**. Otsuka et al. [36] reported that adults who died by suicide had TL shortening and increased mtDNAcn in blood, which is consistent with our findings. However, it should be noted that the MDD + SA/SI group and MDD-SA/SI groups were not significantly different.

A previous meta-analysis in the adult population also revealed that TL shortening was significantly associated with the severity of MDD [28] and that TL shortening was associated with the number of suicide attempts in MDD patients [51]. Another study showed a significantly higher association between TL shortening and suicide in patients with age <50 years; this association was absent in older patients with age >50 years [52], possibly due to the typical TL shortening associated with aging. Previous studies have also reported a significant correlation between TL shortening and higher severity and longer durations of depressive symptoms in adults [23], as well as BAI scores in adult MDD patients [53]. Our study showed a significant correlation between depression severity measured by the clinician-rated CDRS and TL but no correlation with the self-rated BDI-II. However, TL was significantly correlated with the BAI score. A previous study has shown that depressed individuals with anxiety have a higher risk of suicidal ideation and suicide attempts than those without anxiety [54]. Recently, Ford et al. [55] reported that adolescents with current MDD had significantly shorter TL than adolescents who were never diagnosed with MDD, and TL was negatively associated with higher depressive symptom scores. Furthermore, they reported that TL in adolescents with an anxiety disorder was not significantly shorter than in adolescents who were never diagnosed with anxiety disorder; however, TL was negatively associated with higher anxiety symptom scores. This is consistent with our current study and suggests that TL is associated with depressive symptoms and comorbid anxiety, especially suicidality. mtDNAcn was not associated with any of the three measurements, CDRS, BDI-II, or BAI. These results are consistent with the findings in adults of a positive correlation between depression severity and TL, at least with the objective measure of depression.

mtDNAcn was significantly correlated with EA as measured by the CTQ, whereas TL was not. Interestingly, when the group contrasts between the overall MDD and the subgroups versus HC for mtDNA was repeated with EA as a covariate, the results for overall MDD group and the MDD + SA/SI subgroup versus HC for mtDNAcn became non-significant. This raises the possibility that the increased mtDNAcn in MDD patients in general and that associated with the MDD + SA/SI subgroup in particular may be moderated by history of EA and may not be related to depression or suicidality per se. This clearly needs to be replicated.

On our first examination, it would appear that only EA was associated with mtDNAcn, and not other types of abuse (e.g., PA, SA, PN, and EN). However, the lack of association of mtDNAcn with other types of trauma may well be related to a restricted range problem. As evident in Table 1, with the exception of PN, the differences between the HC and either the total MDD sample or the two MDD subgroups were very small. Only EN and PN achieved statistical significance, and the absolute difference of the means for PN was very modest. In adult MDD samples, the CTQ subscale scores are usually significantly higher, particularly for PA. It is unclear exactly why the scores would be lower in an adolescent sample. It is possible that this is due to underreporting of other abuse, particularly physical and sexual abuse, by an adolescent sample. It may also be due to a selection bias, in that adolescents who had experienced higher levels of abuse or their caregivers may have elected not to participate in the study, since the consent form was explicit regarding questions about trauma history. Future studies may need to ascertain trauma history using different methodology.

The mechanisms associated with the shortening of TL are not clearly understood; however, altered HPA axis activity has been considered to play a key role in cellular senescence [12]. It has been shown that TL shortening was associated with greater activity of the HPA axis in children [56] and that increased glucocorticoid concentrations in early life were associated with mitochondrial inefficiency and the shortening of telomeres [57]. Furthermore, glucocorticoid-induced oxidative damage can modify nucleotide sequences within the telomere, which in turn, may accelerate the telomere shortening process. This is supported by a study demonstrating a significant negative correlation between oxidative stress and TL shortening in MDD patients [24] and that cortisol can reduce the transcription of telomere reverse transcriptase, an enzyme responsible for TL gene expression [58]. TL can also be regulated by FK506-binding protein 51, encoded by *FKBP5*, which is an hsp90 co-chaperone. *FKBP5* is activated in response to stress, which inhibits glucocorticoid receptor (GR) translocation, thereby modulating GR sensitivity, a critical element of HPA axis modulation [59, 60]. Several groups, including ours, suggest altered HPA axis activity in suicidal adolescents [61, 62]. In addition, we recently summarized studies showing that epigenetic changes in FKBP5 gene were associated with adolescent depression with ELS [5]. Most recently, Rizavi et al. [63] reported that FKBP5 methylation was significantly decreased, and mRNA expression was significantly increased in the prefrontal cortex and hippocampus, and decreased mRNA expressions, and increased methylations of some GR variants in the prefrontal cortex of teenage suicide completers. These studies indicate that *FKBP5* gene could play an important role in adolescent depression and suicide. Whether *FKBP5* gene is involved with the observed reduced TL shortening in depressed suicidal adolescents, needs further study.

He et al. [64] reported that mtDNAcn was not significantly associated with moderate MDD in young adults, though mtDNAcn was significantly negatively correlated with depressive symptoms. Humphreys et al. [37] also found no association between depressive symptoms in adolescents and mtDNAcn at baseline; however, depressive symptoms at baseline predicted a greater increase in mtDNAcn over a follow-up period of two years. In contrast, a study by Chang et al. [65] reported a significant association between decreased mtDNAcn and MDD in the adult population. Similarly, a postmortem brain study reported lower mtDNAcn in the prefrontal cortex of adult suicide completers compared to healthy controls [36]. Our finding of significantly higher mtDNAcn in the MDD suicidal group exclusively is interesting and suggests that increased mtDNAcn may specifically be associated with suicidality in adolescents.

Our findings of both reduced TL and increased miDNAcn are consistent with recent studies showing telomere shortening can affect mitochondria activity [36, 37, 66]. It has been suggested that TL and mtDNAcn interact with each other, and this interaction could be regulated via the peroxisome proliferator-activated receptor gamma coactivator 1α and 1β (PGC-1α and PGC-1β), master regulators of mitochondrial biogenesis [12, 67]. PGC-1α and PGC-1β activate p53, which plays a key role in regulating cellular senescence via p53/p21^cip1^ pathway [68]. When DNA damage occurs due to telomere erosion, p53, and DNA damage response pathways are activated, which in turn suppress PGC-1α and PGC-1β, leading to mitochondrial dysfunction [69, 70]. In the aging process, telomere shortening increases mitochondria biogenesis. This is mediated via the activation of ataxia telangiectasia-mutated (ATM)-dependent DNA damage, which in turn, stimulates AKT, resulting in PGC-1β-dependent mitochondrial biogenesis and reactive oxygen species generation [71]. TL shortening might be causing mitochondrial dysfunction via ATM-P53-PGC1α/β in adolescent depressed suicidal patients. In a mouse study, Cai et al. [35] found that stress significantly increased the amount of mtDNA and decreased telomere length in saliva and blood. Administration of corticosterone to mice also showed similar changes, suggesting that the hyperactive HPA axis might be influencing these molecular changes. Whether P53/PGC/ATM pathways are involved and work synergistically or independently with the HPA axis in modulating TL and mtDNAcn in adolescent suicidal patients’ needs further study.

Activation of inflammatory pathways has been consistently linked with major depression [72, 73], suggesting that immune dysregulation may play a central role in the pathogenesis of depression. In addition, pro-inflammatory conditions may play an upstream role in suicide-related behavior, and pro-inflammatory states may be independently associated with the risk of suicidality [74–79]. Interestingly, it has been shown that diseases associated with chronic inflammation are also associated with telomere shortening [80–82], suggesting the possibility of an interaction of inflammation and telomere dysfunction and their contribution to disease and progression [83]. In the future, it will be worth pursuing if the observed TL shortening is associated with increased inflammatory markers in adolescent MDD suicidal patients.

A few earlier studies have reported that shorter TL and higher mtDNAcn are associated with ELS in adults [35, 66]. This study reports for the first time that among adolescents increased mtDNAcn, but not TL shortening was significantly correlated with ELS, specifically with emotional abuse. ELS is a known risk factor for MDD and suicidality in both adults and adolescents [5]. It has been reported that sexual, physical, and emotional abuse increases the risk for MDD-emotional abuse being the strongest [84, 85]. Zhang et al. [86] reported that emotional abuse was significantly associated with depression in adolescents via low levels of self-compassion and negative automatic thoughts but not with depression directly. On the other hand, physical abuse was significantly associated with depression directly [86]. Another report also suggested that emotional abuse and neglect were significantly associated with depressive symptoms via low levels of self-compassion in adulthood [87]. Interestingly, mindfulness-based meditation focusing on self-compassion was correlated with longer TL in adults [88–90].

These results, coupled with our findings, raise the possibility that impairment of self-compassion related to emotional abuse may play an essential role in the biological and psychological aspects of the development of MDD via the pathway of cellular senescence. Combined evaluation of self-compassion and cellular senescence biomarkers, such as TL and mtDNAcn, may provide an effective clinical and biological tool for identifying elevated risk of MDD with suicidal ideation in adolescents. Furthermore, mtDNAcn may be more sensitive to environmental factors, including emotional abuse, than other factors in cellular senescence, such as TL, in adolescents.

This present study has some limitations. First, this is a cross-sectional study and cannot clarify the longitudinal relationships between TL, mtDNAcn, and the onset of MDD. Second, our data suggests that there may be a progressive decline in TL and an increase in mtDNAcn in MDD-SA/SI and MDD + SA/SI groups compared to HC group. However, the MDD-SA/SI did not significantly differ from either the HC or MDD + SA/SI groups. This could be due to a relatively small sample size in each group. Third, race was limited to Whites and African Americans. Previous meta-analyses in bipolar patients showed that changes in mtDNAcn were significantly associated with the Asian population [91], prompting them to include more ethnic groups in the study population. Fourth, we did not investigate the epigenetic aspect of TL and mtDNAcn. As we found an association between emotional abuse and mtDNAcn in adolescent MDD subjects, it will be interesting to examine if the observed modifications in TL and/or mtDNAcn are impacted via epigenetic mechanisms. Interestingly, a recent study showed that telomere homeostasis and genomic stability are regulated via m^6^A methylation [92]. Finally, the present study lacks proinflammatory cytokine data. This could have been useful in determining the relationship between the TL shortening and abnormalities in immune functions, as discussed previously. Finally, while the primary comparisons were significant after correction for multiple comparisons, the remaining tests were exploratory and replication is needed.

Altogether, it appears that altered TL and mtDNAcn may play an important role in adolescent depression, particularly in the context of suicidality, and that ELS, particularly emotional abuse, could mediate the differences in mtDNAcn. As mentioned previously, telomeres protect the ends of chromosomes, thereby preventing DNA degradation, recombination, and DNA end fusions. TL shortening thus significantly impacts cellular aging. In addition, telomere length and mitochondrial functions are regulated in a highly shared manner. Given the emerging body of evidence implicating cellular aging in psychiatric disorders, our present study is highly relevant and suggests that both mtDNAcn and TL can serve as biomarkers for suicidality in adolescent depressed subjects and targets for therapeutics. In fact, it has been shown that indices of cellular protection may be involved in the therapeutic mechanisms of psychological treatment for anxiety [93] and that lithium’s mechanism of action is intimately connected with the interdependent regulation of mitochondrial bioenergetics and telomere maintenance [94]. Further longitudinal studies will be needed to test if the abnormalities in TL and mtDNA content in adolescent MDD subjects are the causative factors or precede behavioral changes and whether they can serve as targets of antidepressant action.

### Supplementary information

Supplemental Materials

## Data Availability

All data needed to evaluate the conclusions in the paper are present in the paper or the Supplementary materials.
