# Supplementary material for: Strong associations of telomere length and mitochondrial copy number with suicidality and abuse history in adolescent depressed individuals
Source: Mol Psychiatry. 2023 Sep 21;28(9):3920–9. doi: 10.1038/s41380-023-02263-0 (PMC10730407; doi:10.1038/s41380-023-02263-0)
Supplement: Supplementary file 1 — Supplemental Materials [file 41380_2023_2263_MOESM1_ESM.docx]

**Supplementary Table 1. Correlations between each variable and telomere length and between each variable and mtDNA copy number**

|  | **Telomere length** | | **mtDNA copy number** | |
| --- | --- | --- | --- | --- |
| **Variables** | ***r*** | ***P* value** | ***r*** | ***P* value** |
| **Diagnosis** | -0.36 | 0.008^**^ | 0.35 | 0.009^**^ |
| **Gender** | 0.19 | 0.18 | -0.26 | 0.061 |
| **Race** | 0.21 | 0.12 | 0.097 | 0.49 |
| **Age** | 0.028 | 0.85 | -0.23 | 0.10 |
| **BDI-Ⅱ total** | -0.19 | 0.17 | 0.20 | 0.16 |
| **CDRS total** | -0.36 | 0.010^*^ | 0.13 | 0.36 |
| **BAI total** | -0.29 | 0.039^*^ | 0.14 | 0.32 |
| **C-SSRS PastWeek** | -0.29 | 0.26 | 0.051 | 0.85 |
| **C-SSRS Lifetime** | 0.11 | 0.54 | -0.090 | 0.61 |
| **CTQ total** | -0.26 | 0.056 | 0.24 | 0.089 |
| **CTQ Physical Abuse** | -0.059 | 0.68 | 0.11 | 0.45 |
| **CTQ Physical Neglect** | -0.23 | 0.10 | 0.19 | 0.18 |
| **CTQ Emotional Abuse** | -0.24 | 0.079 | 0.29 | 0.035^*^ |
| **CTQ Emotional Neglect** | -0.23 | 0.095 | 0.20 | 0.16 |
| **CTQ Sexual Abuse** | -0.20 | 0.15 | 0.13 | 0.34 |

The data were analyzed using Spearman's correlation. P <0.05 was considered significant. *p <0.05, **p <0.01. MDD: Major Depressive Disorders; SA: Suicidal Attempt; SI: Suicidal Ideation; BDI: Beck Depression Inventory; CDRS: Children's Depression Rating Scale; BAI: Beck Anxiety Inventory; C-SSRS: Columbia-Suicide Severity Rating Scale; CTQ: Childhood Trauma Questionnaire; mtDNAcn: mtDNA copy number.

**
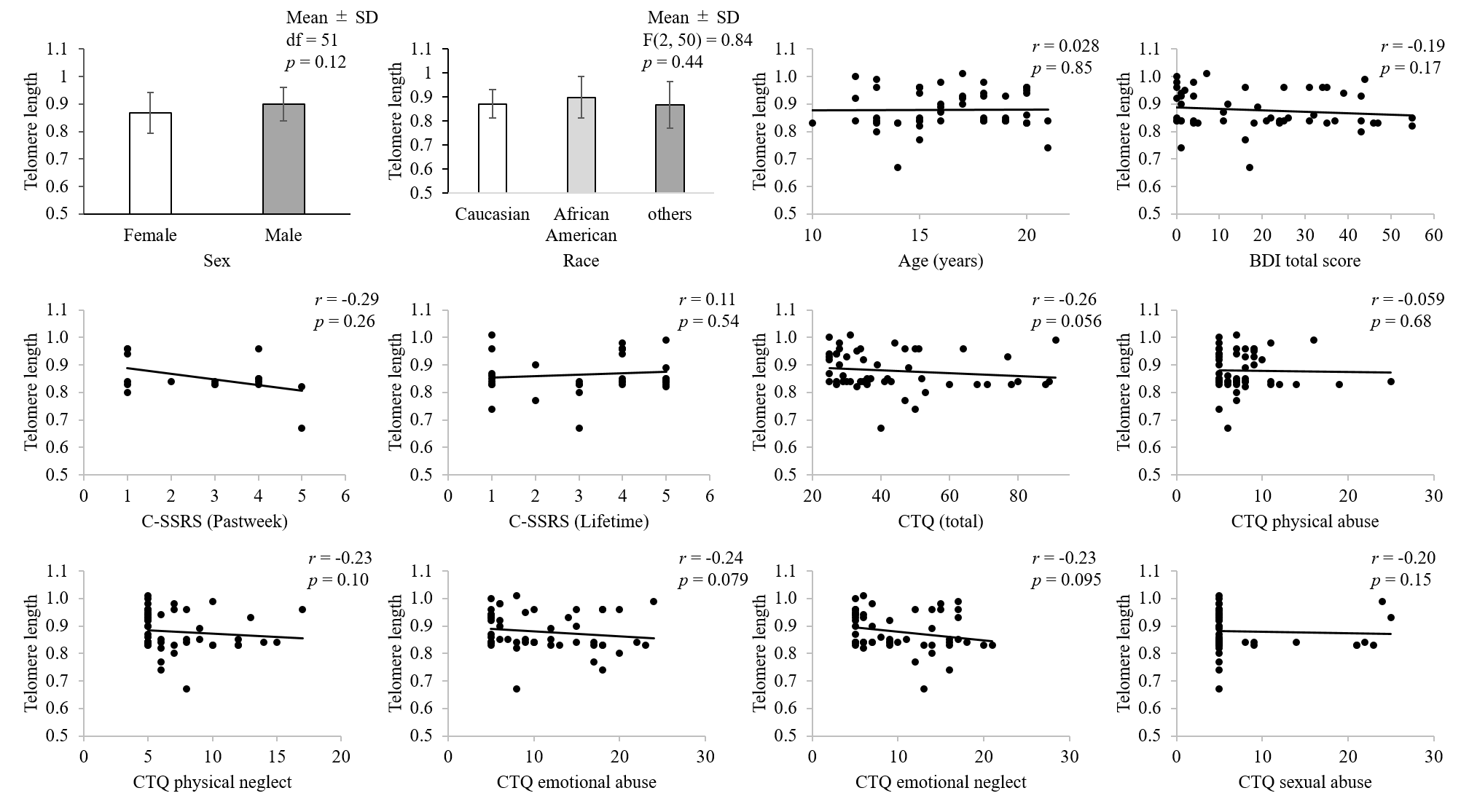
**

**Supplementary Figure 1. Correlations between telomere length and each variable.** There were no significant differences in TL between males and females and between various races. TL was not significantly correlated with, age, BDI-Ⅱ total, C-SSRS past week, C-SSRS Lifetime, CTQ total, CTQ physical abuse, CTQ physical neglect, CTQ emotional abuse, CTQ emotional neglect and CTQ sexual abuse. Spearman's correlation was used. *P* <0.05 was defined as significance. BDI: Beck Depression Inventory; C-SSRS: Columbia-Suicide Severity Rating Scale; CTQ: Childhood Trauma Questionnaire; TL: Telomere Length.

**
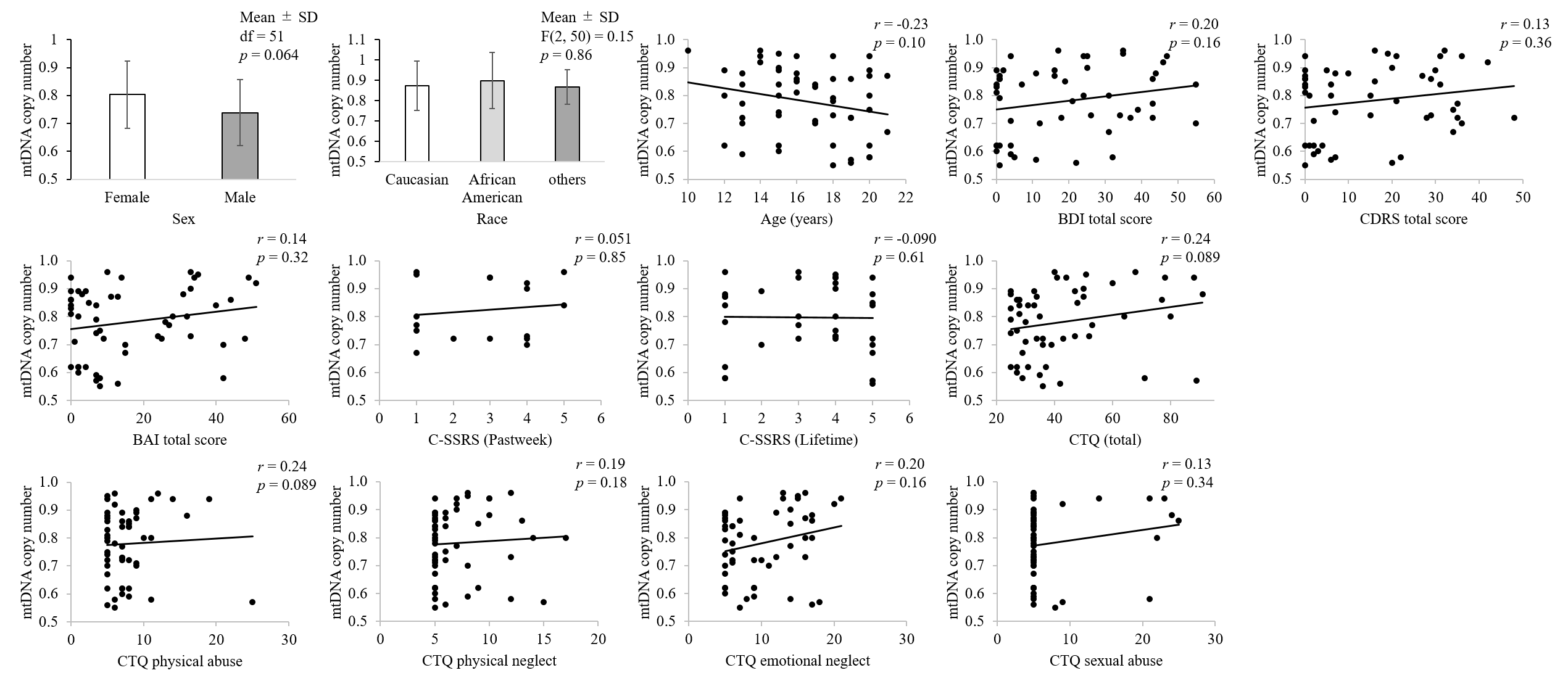
**

**Supplementary Figure 2. Correlations between mtDNA copy number and each variable.** There were no significant differences in mtDNAcn between males and females and between various races. mtDNA copy number was not significantly correlated with age, BDI-Ⅱ total, CDRS total, C-SSRS past week, C-SSRS Lifetime, CTQ total, CTQ physical abuse, CTQ physical neglect, CTQ emotional neglect, and CTQ sexual abuse. Spearman's correlation was used. *P* <0.05 was defined as significance. BDI: Beck Depression Inventory; CDRS: Children's Depression Rating Scale; BAI: Beck Anxiety Inventory; C-SSRS: Columbia-Suicide Severity Rating Scale; CTQ: Childhood Trauma Questionnaire; mtDNAcn: mtDNA copy number.
